# Validation of diffuse correlation spectroscopy measures of critical closing pressure against transcranial Doppler ultrasound in stroke patients

**DOI:** 10.1117/1.JBO.26.3.036008

**Published:** 2021-03-27

**Authors:** Kuan-Cheng Wu, John Sunwoo, Faheem Sheriff, Parisa Farzam, Parya Y. Farzam, Felipe Orihuela-Espina, Sarah L. LaRose, Andrew D. Monk, Mohammad A. Aziz-Sultan, Nirav Patel, Henrikas Vaitkevicius, Maria Angela Franceschini

**Affiliations:** a Massachusetts General Hospital and Harvard Medical School, Optics at Athinoula A. Martinos Center for Biomedical Imaging, Department of Radiology, Charlestown, Massachusetts, United States; b Boston University, Department of Biomedical Engineering, Boston, Massachusetts, United States; c Brigham and Women’s Hospital, Department of Neurology, Boston, Massachusetts, United States; d National Institute for Astrophysics Optics and Electronics, Department of Computational Sciences, Puebla, Mexico; e Brigham and Women’s Hospital, Department of Neurosurgery, Boston, Massachusetts, United States

**Keywords:** critical closing pressure, diffuse correlation spectroscopy, intracranial pressure, ischemic stroke, near-infrared spectroscopy

## Abstract

**Significance**: Intracranial pressure (ICP), variability in perfusion, and resulting ischemia are leading causes of secondary brain injury in patients treated in the neurointensive care unit. Continuous, accurate monitoring of cerebral blood flow (CBF) and ICP guide intervention and ultimately reduce morbidity and mortality. Currently, only invasive tools are used to monitor patients at high risk for intracranial hypertension.

**Aim:** Diffuse correlation spectroscopy (DCS), a noninvasive near-infrared optical technique, is emerging as a possible method for continuous monitoring of CBF and critical closing pressure (CrCP or zero-flow pressure), a parameter directly related to ICP.

**Approach:** We optimized DCS hardware and algorithms for the quantification of CrCP. Toward its clinical translation, we validated the DCS estimates of cerebral blood flow index (${\mathrm{CBF}}_{i}$) and CrCP in ischemic stroke patients with respect to simultaneously acquired transcranial Doppler ultrasound (TCD) cerebral blood flow velocity (CBFV) and CrCP.

**Results:** We found CrCP derived from DCS and TCD were highly linearly correlated (ipsilateral $R^{2}=0.77$, $p=9\times 10^{-7}$; contralateral $R^{2}=0.83$, $p=7\times 10^{-8}$). We found weaker correlations between ${\mathrm{CBF}}_{i}$ and CBFV (ipsilateral $R^{2}=0.25$, p=0.03; contralateral $R^{2}=0.48$, $p=1\times 10^{-3}$) probably due to the different vasculature measured.

**Conclusion:** Our results suggest DCS is a valid alternative to TCD for continuous monitoring of CrCP.

## 1 Introduction

In the healthy brain, and under normal intracranial pressure (ICP), cerebral autoregulation ensures that adequate constant cerebral blood flow (CBF) is maintained over a wide range of arterial blood pressures (ABP).^1^^,^^2^ However, in patients suffering from conditions as shock, stroke, cerebral edema, or traumatic brain injury, their cerebral autoregulation can be impaired such that changes in ABP may lead to cerebral hyperperfusion, hypoperfusion, and ischemia.^3^ If CBF and ICP abnormalities are discovered promptly, therapeutic interventions such as administration of vasoactive agents, osmolar agents, or changes in posture or ventilation can be successfully applied.^4^^–^^6^ Because of the possibility of disrupted autoregulation, blood pressure monitoring alone only marginally helps to assess the impacts of systemic vascular changes to brain perfusion in these patients.

Continuous monitoring of CBF and ICP is needed to optimize the management of critically ill neurointensive care unit (Neuro-ICU) patients and reduce morbidity and mortality.^7^^,^^8^ Current gold standard techniques for CBF and ICP continuous monitoring are invasive, requiring surgical insertion of an intracranial catheter through a hole drilled into the skull.^9^ Because of the invasiveness of the methods and the associated risks of hemorrhage and infection, ICP and CBF monitoring are not done for diagnosis, but only for clinical management in a limited patient population, in cases at high risk for intracranial hypertension.^10^^,^^11^ Development of noninvasive monitoring of CBF and ICP not only will avoid the complications of invasive monitoring in high-risk patients but also will allow inclusion of patients whose risk may be substantial but not enough to justify the invasive procedure. Furthermore, noninvasive measurements would aid in identifying patients who may need invasive monitoring and allow for monitoring patients in critical periods before an invasive sensor can be applied. The problem is that current experimental noninvasive ICP monitoring devices^12^ are suboptimal, operator dependent, or not accurate enough.

Transcranial Doppler ultrasound (TCD) is currently the predominant method used to assess cerebral blood flow velocity (CBFV) and esitimate ICP noninvasively.^13^ TCD measures the velocity of blood inflow in a large cerebral artery such as the middle cerebral artery (MCA), to estimate regional blood flow in the tissue served by this artery with the assumption that the diameter of the insonated vessel remains constant.^14^ TCD can measure both mean and pulsatile blood flow (${\mathrm{pBF}}_{i}$) velocities.

Two analytical methods using the pulsatile features of CBFV obtained by TCD have been proposed to assess ICP.^13^^,^^15^^–^^19^ The first method quantifies a pulsatility index (PI), the ratio of the amplitude of ${\mathrm{pBF}}_{i}$ to the mean blood flow [PI = (systolic flow velocity − diastolic flow velocity)/mean flow velocity]. The TCD-based PI reflects the ICP that influences intracranial compliance and blood flow pulsatility,^15^^,^^18^ and greater blood pressure pulsatility imparts greater CBF pulsatility. Unfortunately, factors such as hypotension and hypocapnia, also influence the value of PI, which limits its specificity and makes it the least accurate TCD-based method for estimating ICP.^13^^,^^20^^,^^21^ The second method quantifies the critical closing pressure (CrCP), the minimal transmural pressure across the vessel wall below which brain vessel collapses and blood flow ceases.^22^^,^^23^ CrCP was first introduced by Burton,^22^ who proposed the use of Laplace’s law to explain the influence of active wall tension on collapsible vessels. The Laplace’s law model assumes the hydrostatic pressure inside the vessel is equal to the wall tension divided by the vessel radius. When the perfusion pressure falls below a certain value, the transmural pressure is not able to counteract the active tension imposed by the vascular smooth muscle layer and the vessel collapse. At this point, blood flow stops, and this perfusion pressure value is defined as CrCP. Inside the skull, CrCP depends on both the vascular wall tension (VWT) and ICP. With TCD, CrCP is obtained from the extrapolated zero flow crossing of the pulsatile components of CBFV and arterial blood pressure (pCBFV and pABP).^15^^,^^18^^,^^24^ CrCP depends on both ICP and VWT.^22^^,^^25^^,^^26^ And for ICP values below 20 mmHg, the influence from VWT in large upstream arteries greatly affects the TCD reading.^19^^,^^27^ This can be one limitation of using TCD-based CrCP values for ICP monitoring; nevertheless, CrCP values can serve as an important biomarker for patients in an ICU needing a prompt customized treatment. In fact, the difference between mean arterial blood pressure (MAP) and CrCP indicates the effective pressure gradient in the brain (or effective cerebral perfusion pressure, ${\mathrm{CPP}}_{\mathrm{eff}}$).^28^^,^^29^ While TCD has proved reliable in assessing CBFV and CrCP, the problem is that it cannot be used continuously for extended periods because of the bulkiness of the ultrasound transducers, the uncomfortable wearability, and the difficulty to maintain constant alignment with the MCA. In addition, a significant proportion of patients do not have a temporal bone window suitable for insonation.^30^

We have previously proposed using diffuse correlation spectroscopy (DCS) instead of TCD to measure CrCP.^31^^–^^33^ DCS is an emerging optical method enabling measurement of an index of blood flow (${\mathrm{BF}}_{i}$) noninvasively and continuously. Similar to near-infrared spectroscopy (NIRS), DCS uses lights to interrogate biological tissues, but, instead of quantifying hemoglobin concentration and oxygenation from the measure of light attenuation, DCS quantifies ${\mathrm{BF}}_{i}$ by measuring the speckle intensity fluctuations generated by the dynamic scattering of moving red blood cells.^34^^–^^36^ In particular, the ability of DCS to quantify changes in cerebral blood flow (${\mathrm{CBF}}_{i}$) has been demonstrated against gold standards both in animal and human studies.^37^^–^^39^ Demonstration of our original idea of using DCS pulsatile cerebral blood flow index (${\mathrm{pCBF}}_{i}$) instead of TCD pCBFV^31^ to quantify CrCP has been tested by other groups against TCD in healthy subjects with a frequency-domain analysis.^40^^,^^41^ In a study on monkeys, a machine learning algorithm based on features in the ${\mathrm{pCBF}}_{i}$ waveform measured on the exposed skull has been used to estimate ICP.^42^ Although these studies found a good correlation between the CrCP estimation and gold standards, the ${\mathrm{pCBF}}_{i}$ signals remain difficult to estimate in humans because of the need of fast acquisition times and the low signal-to-noise ratio (SNR) of DCS devices. We have developed a DCS system able to compute autocorrelation functions at 100 Hz, and, to overcome the low SNR, we have implemented a cardiac gating averaging algorithm, resulting in ${\mathrm{pCBF}}_{i}$ waveforms with high temporal resolution and high SNR. This allow us to better interpret the relationship between pCBFi and pABP, exclude nonlinear components, and provide more robust fitting results. We tested our methodology in stroke patients, which exhibit a wider range of ICP than what can be attained in healthy subjects,^43^ and validated the DCS-derived CrCP against the TCD CrCP estimates.

## 2 Material and Methods

### 2.1 Study Protocol

We recruited acute ischemic stroke patients from the ER and Neurocritical Care Departments at Brigham and Women’s Hospital from May to October 2017. Inclusion criteria included patients affected by acute anterior ischemic strokes with large vessel occlusion, having a National Institute of Health Stroke Score (NIHSS) of 5 or higher within 72 h after last seen well (LSW), and available for DCS and TCD monitoring within 120 h after LSW. We excluded patients who could not tolerate TCD headgear for at least 5 min and patients without sufficient temporal bone windows to obtain reliable TCD readings (Table 1).

**Table 1:** Information about the 14 enrolled acute ischemic stroke patients.

Patient #	Gender	Age	Stroke kind	Stroke side	Admission NIHSS	Area of hemorrhagic infraction (a)	Discharge NIHSS	mRS score @ 30 days follow up
1	Male	52	Ischemic, ICA	Left	13	PH1	15	5
2	Female	31	Ischemic, ICA	Right	14	NB	12	4
3	Male	48	Ischemic, M1	Right	12	HI2	4	4
4	Female	19	Ischemic, M1	Left	11	HI1	16	4
5	Male	73	Ischemic, M2	Right	18	NB	2	2
6	Male	63	Ischemic, M2	Left	23	NB	5	1
7	Female	88	Ischemic, M2	Right	7	NB	6	4
8	Male	83	Ischemic, ICA	Left	7	NB	0	2
9	Male	50	Ischemic, ICA	Left	5	NB	NA	NA
10	Female	66	Ischemic, multiple	Right	13	HI2	2	1
11	Female	47	Ischemic, multiple	Left	9	NB	0	1
12	Female	74	Ischemic, M1	Right	12	HI1	2	0
13	Female	64	Ischemic, M1	Right	16	No	NA	6
14	Male	77	Ischemic, multiple	Right	17	PH2	42	6

a NB: No bleeding. HI1/HI2/PH1/PH2 are types of hemorrhagic transformation. Hemorrhagic infarction (HI) is a petechial infarction without space-occupying effect; Parenchymatous hematoma (PH) is a hemorrhage (coagulum) with mass effect. Subtypes indicate severity; 1 (less severe) or 2 (more severe); mRS, modified Rankin score.

After screening, 14 acute ischemic stroke patients were included in this study (seven women and seven men, mean age of 59.6±19.7 ranging 19 to 88). We simultaneously measured DCS and TCD on these patients for one to three sessions for a total of 23 sessions. The three sessions were within the first 48 h, within 72 to 120 h, and within 144 to 192 h from LSW. The sessions were separated by a 72-h period. Sessions durations were between 5 and 30 min, depending on how long the patient could tolerate the pressure of the TCD headgear.

The protocol was reviewed and approved by the Institutional Review Board (IRB) for Partners Healthcare. Partners IRB follows *Ethical Principles and Guidelines for the Protection of Human Subjects* (Belmont Report). A legally authorized representative willing to have the patient participate in the study signed the written consent.

### 2.2 Instrumentation

**Fig. 1:**
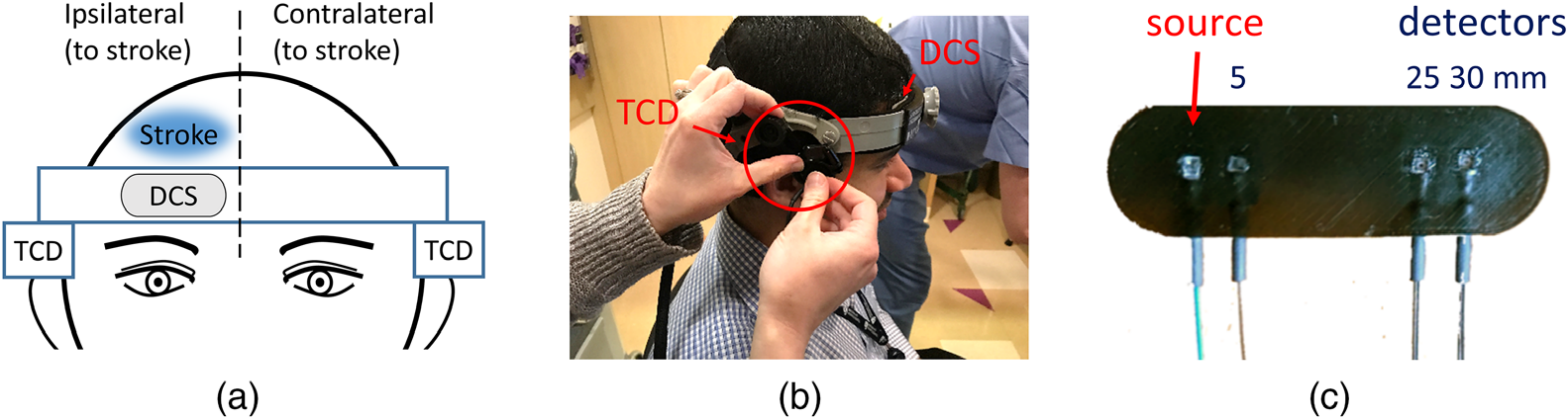
(a) Schematic drawing of locations of DCS probe and TCD transducers. The DCS probe was located on the patient’s forehead, ipsilateral to the stroke; the TCD transducers were positioned over the temporal windows both ipsilateral and contralateral to the stroke. (b) Photo of a coauthor testing the DCS probe and the TCD headgear. The headgear did not only hold the two TCD transducers but also held the DCS probe in contact with the skin. (c) The DCS optical probe consists of one multimode source and four single-mode detector fibers connected to prisms to deliver light to the skin and maintain the low probe profile. Two detectors are connected to the prism at 3 cm from the source to improve SNR.

A certified ultrasound technician trained in transcranial Doppler conducted TCD imaging using two ultrasound transducers (Spencer Technologies) positioned respectively on the left and right temporal windows, to obtain CBFV recording ipsilateral and contralateral to the stroke [Fig. 1(a)]. The transducers were held in place by a headgear, which pressed firmly against the scalp of the patients to maintain constant alignment [Fig. 1(b)].

Conventional DCS systems acquire data at a few Hertz (up to ∼20  Hz), which is not fast enough to resolve ${\mathrm{pBF}}_{i}$. To measure ${\mathrm{pBF}}_{i}$ at high resolution, we have developed a custom DCS device that allows for adjustable integration time and a temporal resolution for photon arrival time of 150 MHz. The system consists of a long coherence length laser at 785 nm (CrystaLaser) and four photon-counting detectors (Excelitas Technologies). The fast acquisition is achieved by a custom-made FPGA-based correlator that allows high-speed transmission of the photon arrival timestamps instead of the intensity temporal autocorrelation ($g_{2}$) curves. To handle this fast data rate, we use a USB3.0 interface (EZ-USB FX3™, Cypress) to stream the data to a computer in real-time. $g_{2}$ can then be postprocessed to the desired time resolution based on the multi-tau algorithm.^44^^,^^45^

To deliver and collect the light to the patient and maintain a good sensor contact with the scalp for an extended period of time, we have developed a low-profile fiber optics probe made with 3D-printed, soft, flexible rubber material [Fig. 1(c)]. For the detectors, we use 5-μm single-mode fibers and for the source a 200-μm multimode fiber. To direct the light perpendicular to the fibers, we use 1.5 mm prisms for both source and detectors. The probe geometry included a short source–detector separation of 5 mm and two large separations of 25 and 30 mm [Fig. 1(c)]. The custom-made probe was positioned on the forehead, under the TCD headgear, on the side ipsilateral to the stroke [Figs. 1(a) and 1(b)] —with an exception in patient 4 session 3, which anyway was excluded in the final analysis because of low SNR. The pressure provided by the headgear against the DCS probe not only secured the skin-sensor contact in place but also reduced the contamination from scalp ${\mathrm{pBF}}_{i}$. The 5-mm separation was used to estimate scalp blood flow index (${\mathrm{BF}}_{i}$). The 25-mm source–detector separation was used to estimate ${\mathrm{CBF}}_{i}$ and ${\mathrm{pCBF}}_{i}$ given that in most patients the data at 30 mm had very low SNR.

In five patients, ABP was continuously acquired via an arterial line that was placed for medical reasons. In all other patients, we acquired ABP noninvasively with a Finapres Nova device (Finapres Medical Systems, Netherland). Finapres-derived ABP was calibrated using the four systolic and diastolic ABP values recorded in the hospital records and closest to the time of our measurement.

ABP and both ipsilateral and contralateral TCD data were coregistered with DCS auxiliary inputs sampled at 50 kHz.

### 2.3 Data Processing

We first generated DCS ${\mathrm{BF}}_{i}$ time-traces at 0.2 Hz by fitting $g_{2}(\tau )$ every 5 s to identify and remove motion artifacts. The identified segments were removed from DCS, TCD, and ABP time-traces.

**Fig. 2:**
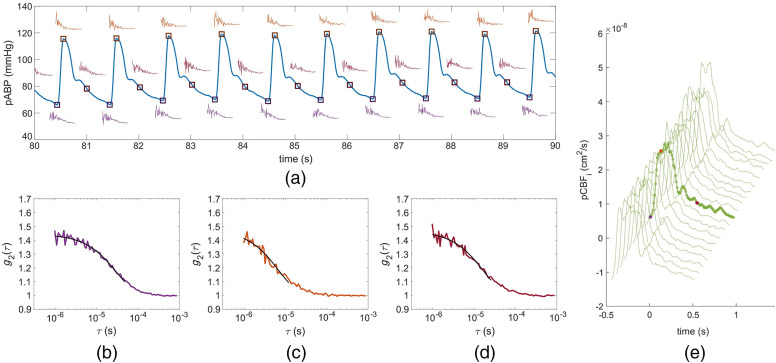
(a) The small graphs along the pABP signal are the instantaneous $g_{2}$ curves at 100 Hz at three times along the arterial pulsation in a subject. (b)–(d) Corresponding $g_{2}$ obtained by averaging 50 instantaneous $g_{2}s$ over 50 heartbeats at the three points shown in (a), (b) diastolic end (purple); (c) systolic peak (orange); and (d) at a point during diastole runoff (red). (e) ${\mathrm{pCBF}}_{i}$ waveforms throughout the subject’s 20 min measurement session. The x and y axis values are for the highlighted waveform. The color-marked data points are the ${\mathrm{BF}}_{i}$ corresponding to the $g_{2}s$ in (b)–(d). Systolic ${\mathrm{CBF}}_{i}$ is about five times higher than diastolic ${\mathrm{CBF}}_{i}$, and this can be clearly seen in the faster $g_{2}$ decay at (c) the systolic peak.

To resolve the ${\mathrm{pBF}}_{i}$, we computed intensity temporal autocorrelation functions at 100 Hz using a moving average of 60 ms of data. With such a short integration time, the photon count is too low for fitting $g_{2}$ and recovering ${\mathrm{BF}}_{i}$. To overcome this issue, we averaged $g_{2}(\tau )$ curves at the same point in the cardiac cycle over 50 heartbeats (as a cardiac gating averaging method). Each heartbeat was identified using the ABP signal, found by the diastolic end → systolic peak → diastolic end pressures. Each averaged $g_{2}$ was fitted to the semi-infinite correlation diffusion equation^46^ using fixed optical properties to obtain ${\mathrm{BF}}_{i}$. In the calculation for all subjects we assumed $\mu_{a}=0.17\;{\mathrm{cm}}^{-1}$ and ${\mu_{s}}^{\prime }=8.8\;{\mathrm{cm}}^{-1}$.^47^ While the optical properties values affect the absolute ${\mathrm{CBF}}_{i}$ and cerebral vascular resistance values, they have no impact on relative ${\mathrm{CBF}}_{i}$ changes and on CrCP estimates.^32^ Finally, we resolved an average pulsatile ${\mathrm{pCBF}}_{i}$ waveform every 50 heartbeats (Fig. 2). This process was repeated for the whole duration of the measurement. Details of the CrCP data processing algorithm are reported in the Supplemental Materials.

The coregistered analog signals, ABP and TCD-based blood flow velocity, were downsampled to 100 Hz. The same averaging algorithm used for DCS was applied to these signals to generate pABP and pCBFV waveforms.

**Fig. 3:**
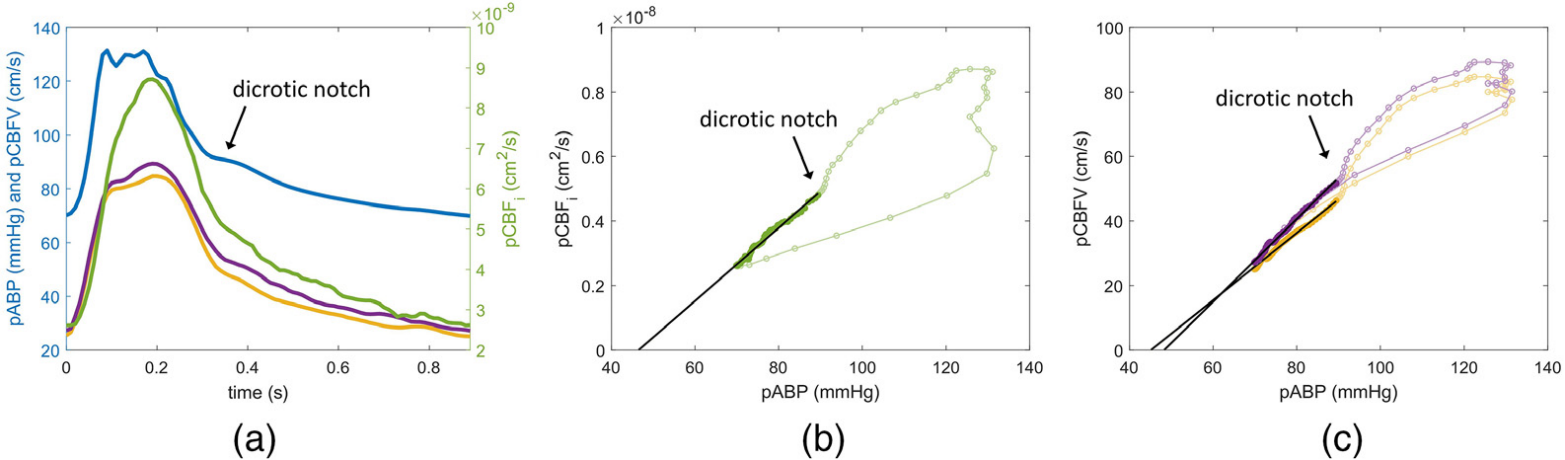
(a) Examples of pABP (blue), ${\mathrm{pCBF}}_{i}$ (green), and pCBFV (yellow, ipsilateral; purple, contralateral) waveforms derived from an average of 50 heartbeats on a representative patient (patient 07-2). The y-axis on the left is for the pABP and pCBFV, whereas the right axis is for ${\mathrm{pCBF}}_{i}$. (b) Scatterplot of ${\mathrm{pCBF}}_{i}$ versus pABP and fit to obtain ${\mathrm{CrCP}}_{\mathrm{DCS}}$. (c) Scatterplot of pCBFV (yellow: ipsilateral, purple: contralateral) versus pABP and ${\mathrm{CrCP}}_{\mathrm{TCD}}$ fits.

Temporal lags between blood pressure and blood flow signals were rectified, prior to fitting for CrCP, by aligning the pulsatile waveforms of pCBFV and ${\mathrm{pCBF}}_{i}$ to pABP using the diastolic end pressure points [see Fig. 3(a) for an example of resulting alignment].

To calculate CrCP, we used the linear regression approach between pABP and pulsatile cerebral blood flow (pCBF, indicating either pCBFV or ${\mathrm{pCBF}}_{i}$). By assuming a single resistor model, the pressure-flow relationship can be written as^22^^,^^24^

pCBF=(pABP−CrCP)/CVR, (1)

where CVR is the cerebrovascular resistance (CVR), defined as

CVR=ΔpABP/ΔpCBF. (2)

CrCP, with pCBF versus pABP relationship, is obtained by linearly extrapolating the data to the pABP-axis intercept. However, as shown in Figs. 3(b) and 3(c), for both DCS and TCD, the scatterplots against pABP form a hysteresis loop during the systole phase. The hysteresis is in part due to the non-perfect alignment between the pABP and pCBF signals, and in part due to different blood vessel compliances seen by each measurement device (e.g., finger versus head), especially during the systole phase when pressure and flow change more rapidly. Hence, to estimate ${\mathrm{CrCP}}_{\mathrm{DCS}}$ and ${\mathrm{CrCP}}_{\mathrm{TCD}}$, we considered only the diastolic runoff part of the signal. Robust regression (function robustfit in Matlab, MathWorks) was used to fit for CrCP instead of least-square linear fit to avoid the impact of possible outliers, such as the early systolic upstroke and fluctuating dicrotic notch.

Using Eq. (2), from the fitted relationship between ${\mathrm{pCBF}}_{i}$ (or pCBFV) and pABP, we also derived the CVR, defined as the inverse of the slope between the runoff parts of pABP and pCBF.

To calculate CrCP, we also considered a frequency domain approach proposed by Aaslid^23^^,^^48^^,^^49^ and adopted for DCS by Baker et al.^40^ This method is also based on the single resistor model mentioned above. The difference is that CrCP is calculated using the frequency component extracted from the signal, whereas assuming the impedance, CVR, is constant across the used frequencies. The relationship can be rewritten as:

pCBF(f)=(pABP(f)−CrCP(f))/CVR. (3)

Assuming CrCP is also a constant, meaning its nonzero frequency components are equal to zero, we obtain^23^

$$\mathrm{CrCP}=\mathrm{pABP}(0)-\frac{\mathrm{pABP}(f)}{\mathrm{pCBF}(f)}\mathrm{pCBF}(0),$$ (4)

where pABP(0) is the mean arterial pressure, and pCBF(0) is the mean CBF. pABP (f) and pCBF(f) represent the amplitude of the signals at the frequency f. Conventionally, the first harmonic of heart rate is used because with high amplitude it provides a higher SNR.

## 3 Results

Fourteen subjects and 20 sessions are included in this work. Three DCS sessions were excluded due to poor SNR (patient 01-2, patient 04-3, and patient 14-3). In addition, because of low SNR in TCD signal, we excluded one contralateral TCD from the dataset (patient 09-1). One ipsilateral TCD dataset was not acquired (patient 03-2). The resulting 20 sessions have an average duration of 16.7 min, with a standard deviation of 3.7 min.

### 3.1 Pulsatile Cerebral Blood Flow Index

Figure 3(a) shows the average pulsatile waveforms pABP, ${\mathrm{pCBF}}_{i}$, and ipsilateral and contralateral pCBFV, over the 50 heartbeats of a representative subject. While shape and features of the pulsatile waveform vary considerably across subjects, within the same subject, similar morphological features, such as the shape of the systolic peak and the dicrotic notch, are visible across modalities.

${\mathrm{pCBF}}_{i}$ shows the largest pulsatile amplitude compared to pulsatile TCD flow velocity and pulsatile ABP. Systolic ${\mathrm{pCBF}}_{i}$ signal is in average 217.8±91.7% higher than the diastolic end ${\mathrm{pCBF}}_{i}$ (n=20); based on two-sample t-test, ${\mathrm{pCBF}}_{i}$ amplitude was statistically significantly larger than that pCBFV ipsilateral 139.8±37.3% [n=19, t(25)=−3.51, p=0.0017], pCBFV contralateral 118.7±46.9% [n=19, t(29)=−4.28, $p<2\times 10^{-4}$], and pABP 97.7±27.3% [n=20, t(22)=−5.61, $p<2\times 10^{-5}$) amplitudes. Variables were tested for normality and were found normal (Kolmogorov–Smirnov: pCBFi D=0.11, p=0.94; ipsilateral pCBFV D=0.20 p=0.41, contralateral pCBFV D=0.19, p=0.45; pABP D=0.16, p=0.64).

Figures 3(b) and 3(c) show the pressure-flow relationship obtained from pABP, ${\mathrm{pCBF}}_{i}$, and pCBFV in a representative subject. The hysteresis around the systolic peak is minimized by optimizing the alignment but still large in this case. Only the data points after the dicrotic notch are used to derive CrCP with DCS and TCD. Because the features of the pulsatile waveform and the hysteresis were quite different across subjects, the dicrotic notch was manually defined for each subject.

### 3.2 Critical Closing Pressure

**Fig. 4:**
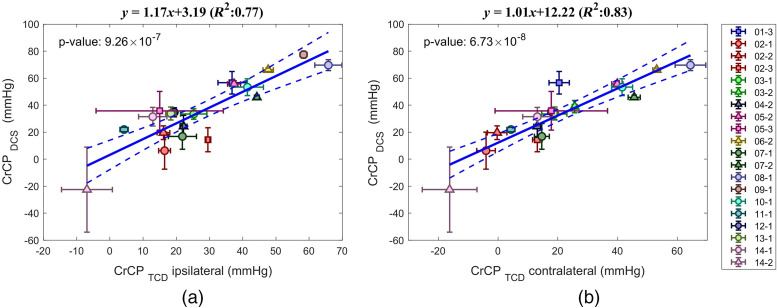
DCS versus TCD-based mean and standard deviation of CrCP. (a) TCD ipsilateral to the stroke, (b) TCD contralateral to the stroke. Different subjects are labeled with different colors. Session 1, circle; session 2, triangle; and session 3, square. Dashed lines mark the confidence interval of the linear regression. Removing the negative point only slighty affect the correlations [(a) $R^{2}=0.71$, (b) $R^{2}=0.81$].

Figure 4 shows the scatterplots of the average CrCP obtained with DCS and TCD for each subject and each session using the linear regression approach. We found strong correlation between ${\mathrm{CrCP}}_{\mathrm{DCS}}$ and ${\mathrm{CrCP}}_{\mathrm{TCD}}$. For ${\mathrm{CrCP}}_{\mathrm{DCS}}$ and ipsilateral ${\mathrm{CrCP}}_{\mathrm{TCD}}$, the coefficient of determination was $R^{2}=0.77$ (slope of 1.17), $p=9.3\times 10^{-7}$ [Fig. 4(a)]; for ${\mathrm{CrCP}}_{\mathrm{DCS}}$ and contralateral ${\mathrm{CrCP}}_{\mathrm{TCD}}$ we found $R^{2}=0.83$ (slope of 1.01), $p=6.7\times 10^{8}$ [Fig. 4(b)]. As expected, the correlation between contralateral and ipsilateral ${\mathrm{CrCP}}_{\mathrm{TCD}}$ was also strong with $R^{2}=0.86$ (slope of 1.11) $p=2.8\times 10^{-8}$ (see Fig. S1(a) in the Supplemental Materials).

The frequency-domain method also showed a positive linear relationship between ${\mathrm{CrCP}}_{\mathrm{DCS}}$ and ${\mathrm{CrCP}}_{\mathrm{TCD}}$ with $R^{2}=0.74$ with $p=1.96\times 10^{-6}$, and $R^{2}=0.78$ with $p=6\times 10^{-7}$ (ipsilateral and contralateral, respectively, see Fig. S2 in the Supplemental Materials). The slope was more inconsistent (1.52 and 0.92, ipsilateral and contralateral, respectively) than with the linear regression method.

There is a good agreement between CrCP derived from linear-regression and frequency domain methods (for DCS, the $R^{2}=0.90$, slope of 1.23, p-value of $1.8\times 10^{-10}$; for TCD ipsilateral to the stroke, $R^{2}=0.85$, slope of 0.90, p-value of $2.2\times 10^{-8}$; for TCD contralateral to the stroke, $R^{2}=0.69$, slope of 1.15, p-value of $1.1\times 10^{-5}$ (see Fig. S3 in the Supplemental Materials).

### 3.3 Pulsatility Index

PI, calculated as PI = (systolic CBF − diastolic CBF)/mean CBF, between DCS and TCD showed weak correlation [see Figs. S4(a) and S4(b) in the Supplemental Materials]. It showed no correlation with ipsilateral TCD ($R^{2}$ of 0.10, p=0.20) and weak correlation with contralateral TCD ($R^{2}$ of 0.30, p=0.015. ${\mathrm{PI}}_{\mathrm{DCS}}$ values were in general higher than ${\mathrm{PI}}_{\mathrm{TCD}}$ values. Also within TCD, between contralateral and ipsilateral to the stroke measurements, the relationship was relatively weak with $R^{2}$ of 0.51 and $p=8\times 10^{-4}$ [see Fig. S4(c) in the Supplemental Materials].

### 3.4 Cerebral Blood Flow and Cerebrovascular Resistance

**Fig. 5:**
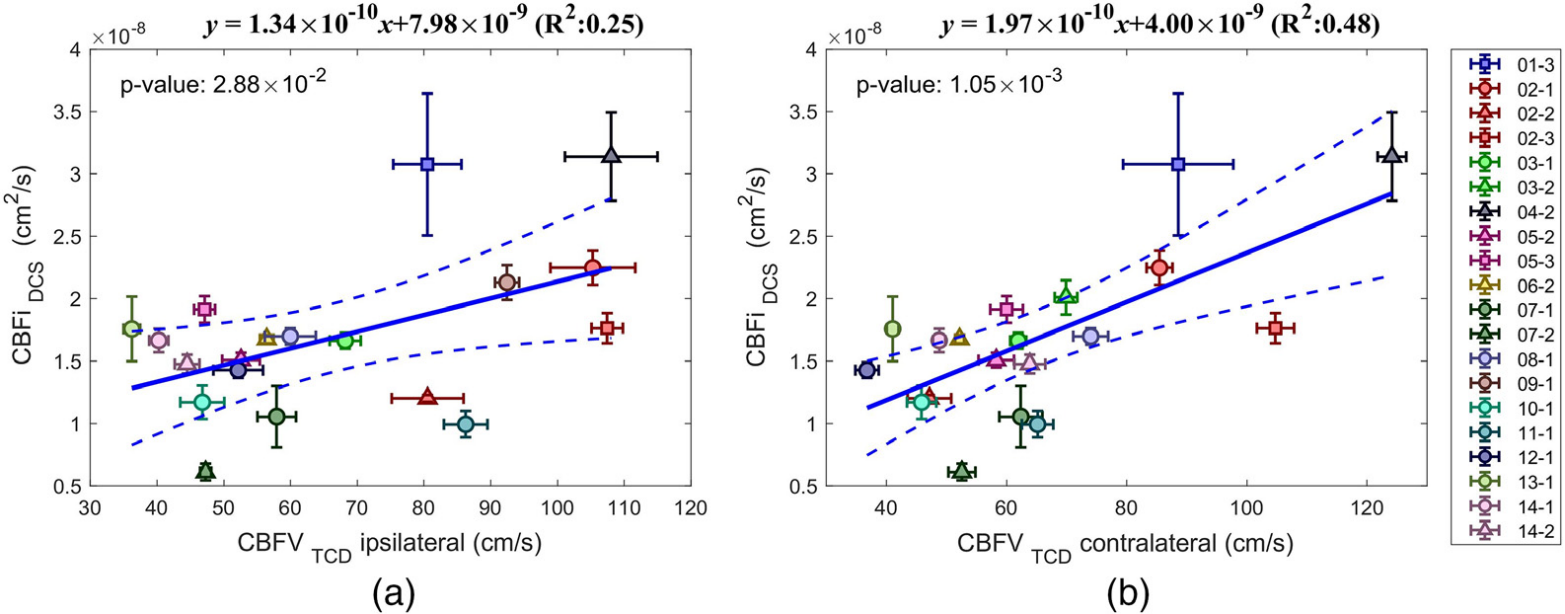
DCS versus TCD-based mean and standard deviation of CBF. (a) TCD ipsilateral to the stroke, (b) TCD contralateral to the stroke. Different subjects are labeled with different colors. Session 1, circle; session 2, triangle; and session 3, square. Dashed lines mark the confidence interval of the linear regression.

A positive correlation was found between mean ${\mathrm{CBF}}_{i}$ and mean CBFV with $R^{2}=0.25$ and $p=2.9\times 10^{-2}$ for the ipsilateral CBFV [Fig. 5(a)] and $R^{2}=0.48$ and $p=1\times 10^{-3}$ for contralateral CBFV [Fig. 5(b)]. The correlation between contralateral and ipsilateral CBFV was stronger with $R^{2}=0.66$, $p=5\times 10^{-5}$ and slope of 0.78 [see Fig. S1(b) in the Supplemental Materials].

**Fig. 6:**
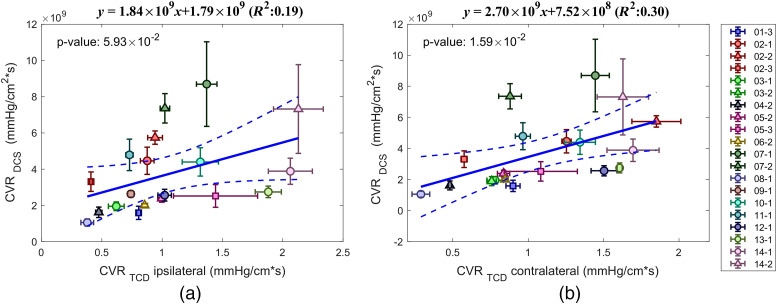
DCS versus TCD-based mean and standard deviation of CVR. (a) TCD ipsilateral to the stroke and (b) TCD contralateral to the stroke. Different subjects are labeled with different colors. Session 1 circle, session 2 triangle, and session 3 square. Dashed lines mark the confidence interval of the linear regression.

Using pCBF and pABP, we derived CVR. A weak positive correlation was found between ${\mathrm{CVR}}_{\mathrm{DCS}}$ and ${\mathrm{CVR}}_{\mathrm{TCD}}$ with $R^{2}=0.19$ and p=0.06 for ipsilateral TCD; $R^{2}=0.30$ and p=0.016 for contralateral TCD (Fig. 6). Instead, the correlation between contralateral and ipsilateral TCD derived CRV was stronger with $R^{2}=0.60$, $p=2\times 10^{-4}$ and slope of 0.66 [see Fig. S1(c) in the Supplemental Materials].

While CVR across modality did not correlate, we found a strong correlation between ${\mathrm{CVR}}_{\mathrm{DCS}}$ and ${\mathrm{CPP}}_{\mathrm{DCS}}$, defined as $\mathrm{MAP}\text{-}{\mathrm{CrCP}}_{\mathrm{DCS}}$ (see Fig. S5 in the Supplemental Materials).

### 3.5 Stroke Parameters Correlations

Finally, we explored additional correlations with clinical parameters relevant to stroke. We found that the Alberta Stroke Program Early CT Score (ASPECTS), a standardized 10-point scale characterizing CT head findings during acute stroke evaluation, showed a strong inverse relation with CBF (DCS: $R^{2}=0.53$, p<0.001; ipsilateral TCD: $R^{2}=0.52$, p<0.001; contralateral TCD: $R^{2}=0.61$, p<0.001). Infarct volume at 24 h presented mild inverse correlations with CrCP (DCS: $R^{2}=0.20$, p<0.05; ipsilateral TCD: $R^{2}=0.24$, p<0.05) as well as positive correlation with CVR (ipsilateral TCD: $R^{2}=0.28$, p<0.05). NIHSS scores and midline shift due to edema at 48 h did not show any effect over CrCP, CVR, or MAP. Hypertension showed a significant relation to ipsilateral CBFV measured with TCD (Mann–Whitney U: Z=2.19, p<0.05), as well as influencing DCS-measured ${\mathrm{CBF}}_{i}$, but not reaching significance. CrCP was also influenced by hypertension, but again it did not reach significance.

Other parameters also correlated with our cerebral measures and seems to play a factor. Age-influenced CBF-higher age associated to lower CBF- (DCS: $R^{2}=0.28$, p<0.05; ipsilateral TCD: $R^{2}=0.73$, p<0.001; contralateral TCD: $R^{2}=0.37$, p<0.01) and mildly influenced CVR—higher age associated to higher CVR—(ipsilateral TCD: $R^{2}=0.26$, p<0.05). The hematocrit mildly correlated positively with CrCP estimated with DCS ($R^{2}=0.19$, p=0.06). MAP also mildly correlated with CrCP (DCS: $R^{2}=0.22$, p<0.05; ipsilateral TCD: $R^{2}=0.23$, p<0.05) and with CBFV (contralateral TCD: $R^{2}=0.24$, p<0.05). Average body temperature correlated inversely with CrCP (DCS: $R^{2}=0.36$, p<0.01; ipsilateral TCD: $R^{2}=0.49$, p<0.01; contralateral TCD: $R^{2}=0.35$, p<0.05).

We do not have sufficient data at this point to make a strong statement about these correlations.

## 4 Discussion

In this study, we acquired ${\mathrm{pCBF}}_{i}$ on 14 acute stroke patients with our custom-built fast DCS system. We developed an algorithm to resolve the ${\mathrm{pCBF}}_{i}$ waveform at high resolution (100 Hz) by cardiac gating and averaging the temporal autocorrelation functions $g_{2}$ over 50 heartbeats. Using the pCBF waveform and the corresponding averaged pulsatile ABP, we derived CrCP. CrCP was calculated by linearly fitting the diastolic runoff of the pulsatile pressure-flow relationship and by extrapolating to the x-axis intercept. DCS-derived CrCP was compared with simultaneously acquired TCD-derived CrCP. We found a statistically significant correlation between the two (ipsilateral $R^{2}=0.77$, $p=9\times 10^{-7}$; contralateral $R^{2}=0.83$, $p=7\times 10^{-8}$), indicating that the DCS method is a validated alternative to TCD in deriving CrCP and has the potential to monitor CrCP noninvasively in human subjects at the bedside for extended periods of time and in patients without adequate bone windows.

DCS ${\mathrm{pCBF}}_{i}$ was measured over the forehead ipsilateral to the stroke while TCD pCBFV was measured through the temporal window in the MCA ipsilateral and contralateral to the stroke. We found a stronger correlation of ${\mathrm{CrCP}}_{\mathrm{DCS}}$ with the contralateral ${\mathrm{CrCP}}_{\mathrm{TCD}}$, probably because of the noisier TCD signal on the stroke side. The linear relationship had a slope close to 1 while having a non-zero intercept indicating that ${\mathrm{CrCP}}_{\mathrm{DCS}}$ was about 8 to 13 mmHg higher than ${\mathrm{CrCP}}_{\mathrm{TCD}}$. This difference is probably attributed to the different VWT of the vessels measured: MCA for TCD^14^ and cortical microvessels for DCS.^50^^,^^51^ MCA is a large vessel in the subarachnoid space at the skull base, the small vessels in the parenchyma measured by DCS are much more fragile and the CrCP estimation with DCS may be much more relevant to cerebral physiology. On the other end, MCA represents the whole MCA territory while the DCS measure is very local so DCS estimates of CrCP may have limitation due to the focality of the measure.

Another source of the discrepancy could be the inflow pressure difference between the two. MCA is more upstream and thus its blood pressure is higher and closer to the systemic blood pressure. Baker et al.,^40^ citing an animal study (rat),^52^ suggested that mean arterial pressure at the entrance of the arteriole compartment is 40% lower than the systemic blood pressure. Assuming the waveform is evenly scaled, they corrected CrCP by multiplying the result by 0.6. Since the correct factor for humans is unknown, we did not used this facor. If the 0.6 factor is applied, the coefficient of determination would not change, whereas the slope and the intercept will both be scaled by the 0.6 factor, making them farther from 1.

To achieve sufficient SNR in determining ${\mathrm{pCBF}}_{i}$ we had to average 50 heartbeats, which provided approximately 1 CrCP value per minute. This slow temporal resolution should not be a problem since CrCP is not expected to change fast and intervention within minutes is acceptable.

The alignment between pABP and ${\mathrm{pCBF}}_{i}$ (or pCBFV) waveforms was obtained by considering the diastolic end-systolic peak-diastolic end pressures. This method was easy to apply and was robust against the difference in the waveform features between modalities. We also tested the cross-correlation method; however, differences in the shape of the systole waveforms made cross-correlation less robust on aligning pCBF with pABP than the diastolic end-systolic peak-diastolic end pressures method.

As shown in Figs. 3(b) and 3(c), for both DCS and TCD, the scatterplots against pABP during the systole phase form a hysteresis loop. We believe the hysteresis is mostly due to the different blood vessel compliances seen by each measurement modality (e.g., finger versus head). The single resistor model can be used to describe the pressure–flow relationship only at low frequency.^22^^,^^24^ As described in the Windkessel model,^53^ at high frequency to describe the compliance of a blood vessel in addition to a resistor, we need to add a capacitance component.^54^ The capacitors in the circuit cause nonlinear changes in flow as the pressure changes. The high-frequency components take place during the systole phase and result in the nonlinear hysteresis loop. The linear behavior is limited to the low frequency diastolic runoff of the cardiac cycle when the changes in pressure and flow are slower and a single resistor model is sufficient to describe a blood vessel compliance. Hence, to estimate ${\mathrm{CrCP}}_{\mathrm{DCS}}$ and ${\mathrm{CrCP}}_{\mathrm{TCD}}$, we considered only the diastolic runoff part of the signal.

The frequency-domain method has the advantage that it does not require signals alignment, but it cannot selectively exclude the data during the high frequency systole phase. Using the frequency domain method, the correlation between ${\mathrm{CrCP}}_{\mathrm{DCS}}$ and ${\mathrm{CrCP}}_{\mathrm{TCD}}$ (see Fig. S2 in the Supplemental Materials) is lower than with the linear regression method, with slopes futher from unity. By applying the 0.6 correction factor, all the slopes become lower than 1 with the slope between ${\mathrm{CrCP}}_{\mathrm{DCS}}$ and ${\mathrm{CrCP}}_{\mathrm{TCD}}$ contralateral equal to 0.42.

As expected, the linear regression and frequency domain methods show relatively good correlations ($R^{2}$ of 0.90 for DCS, 0.85 for ipsilateral TCD and 0.69 for contralateral TCD; see Fig. S3 in the Supplemental Materials), but in general the frequency-domain method provides lower CrCP estimates.

We believe the lower CrCP estimates and the lower $R^{2}$ when comparing DCS with TCD CrCP are due to the inclusion of the high frequency systole phase data into the frequency-domain CrCP calculations.

Mean cerebral blood flow (mean ${\mathrm{CBF}}_{i}$ and mean CBFV) showed positive correlation between DCS and TCD, albeit the correlation was weaker compared to that of CrCP. (Fig. 5) This can be due to the fact DCS and TCD do not measure the same physiological parameters: TCD measures blood flow velocity while DCS measures an index proportional to blood flow^50^^,^^51^ and target different vessels: TCD measured MCA, whereas DCS measured cortical microvasculature. Lastly, there are factors that play a role in determining the absolute values for each method. For TCD, the angle between the sound wave propagation direction and the arterial blood vessel affects CBFV values; for DCS, the absolute ${\mathrm{CBF}}_{i}$ value depends on the optical properties of the illuminated tissue, as well as the density and the average radius of the vessels.^50^ In this work, we have used constant absorption and scattering coefficients across subjects. In reality, we expect differences in these parameters due to vessel density, average vessel radius, scalp thickness, and the distance between the brain and the scalp. To measure brain optical properties in adult subjects, frequency-domain or time-domain near-infrared methods are needed in combination with multilayer models, and preferably with a known thickness of the layers. Instead, CrCP is independent of the assumed optical properties. This is because CrCP is defined as the pressure at the pABP intercept where pCBF goes to zero and derived by a relative change independent of the absolute values of pCBF. This suggests that absolute values of CrCP can be used to compare across subjects.

Optical properties assumptions also affect the CVR values. For CVR, we did not find significant correlation between DCS and TCD. Differences in the resistance between MCA and small cortical vessels may further increase these differences.

Within a modality, we obtained the expected good agreement between CVR and CPP (Fig. S5 in the Supplemental Materials), further suggesting the CVR differences are due to the different vessel measured with the two methods, not due to the CVR calculations per se.

The PI shows low to moderate correlation between DCS and TCD ($R^{2}$ of 0.1 to 0.3) with ${\mathrm{PI}}_{\mathrm{DCS}}$ substantially higher than ${\mathrm{PI}}_{\mathrm{TCD}}$ (Fig. S4 in the Supplemental Materials). This is expected since CBFV is not a direct measurement of velocity, but an integration of velocity over the cross-section of the vessels.^55^ The expended cross-sectional area during the systole phase contributes to the higher pulsation in the blood flow, which contribute to the higher value of ${\mathrm{PI}}_{\mathrm{DCS}}$. In addition, the target vessels are not the same between the DCS and TCD.

Scalp ${\mathrm{pBF}}_{i}$ can contaminate DCS signals and affect the CrCP values. In our case, the pulsatile scalp blood flow component was suppressed by the TCD headgear, which applied strong pressure to the sensor and the scalp of the subjects. Because of that, ${\mathrm{pCBF}}_{i}$ at short separation (5 mm) provided unrealistic results, which varied depending on the pressure applied. The validity of the method when the pressure is not applied to the sensor needs to be tested.

## 5 Conclusion

Noninvasive monitoring of CrCP may provide an opportunity for prompt and optimal management of patients with neurocritical care-related conditions and associated complications. In this work, we validated DCS-derived CrCP against TCD-based CrCP and demonstrated they are comparable to each other. These results prove that DCS is an attractive alternative to TCD for noninvasive CrCP monitoring, with additional benefits that are native to DCS. Future work needs to validate DCS CrCP against invasive ICP, to determine whether CrCP can be used as a proxy for ICP.

## Code, Data, and Materials Availability

Data will be provided upon request.
