# Supplementary material for: Validation of diffuse correlation spectroscopy measures of critical closing pressure against transcranial Doppler ultrasound in stroke patients
Source: J Biomed Opt. 2021 Mar 27;26(3):036008. doi: 10.1117/1.JBO.26.3.036008 (PMC7998065; doi:10.1117/1.JBO.26.3.036008)
Supplement: Supplementary file 1 [file JBO_026_036008_SD001.pdf]

Supplemental Material

1. Supplemental figures

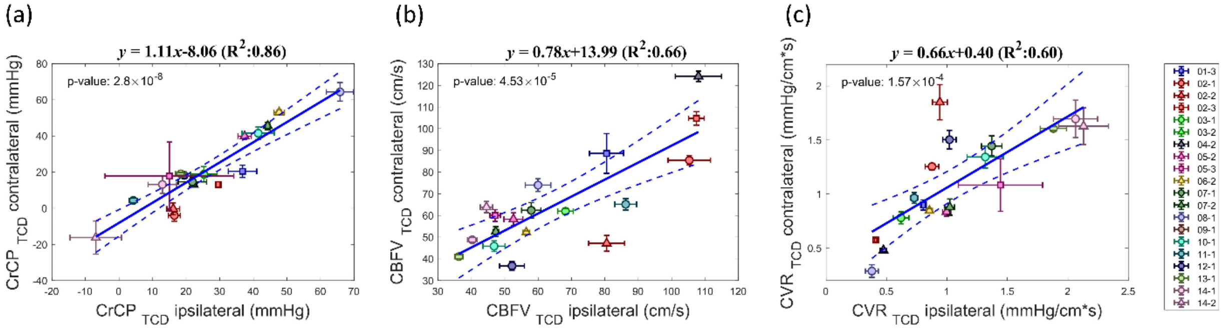

Figure S1. TCD -based mean and standard deviation of CrCP, CBFV, and CVR. Comparisons across contralateral and ipsilateral sides to stroke. Different subjects are labelled with different colors. Session 1 circle, session 2 triangle, session 3 square. Dashed lines mark the confidence interval of the linear regression.

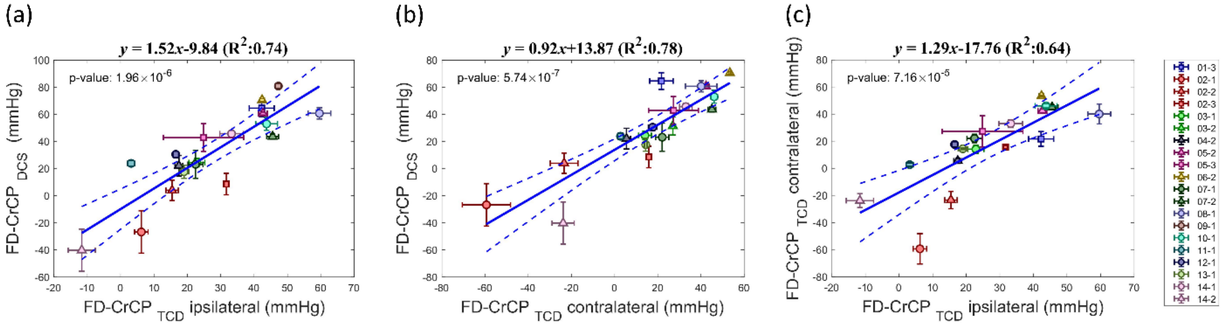

Figure S2. Frequency-domain derived CrCP (FD-CrCP) mean and standard deviation of a) DCS vs. TCD ipsilateral to the stroke, b) DCS vs. TCD contralateral to the stroke. c) TCD ipsilateral vs. TCD contralateral to the stroke. Different subjects are labelled with different colors. Session 1 circle, session 2 triangle, session 3 square. Dashed lines mark the confidence interval of the linear regression.

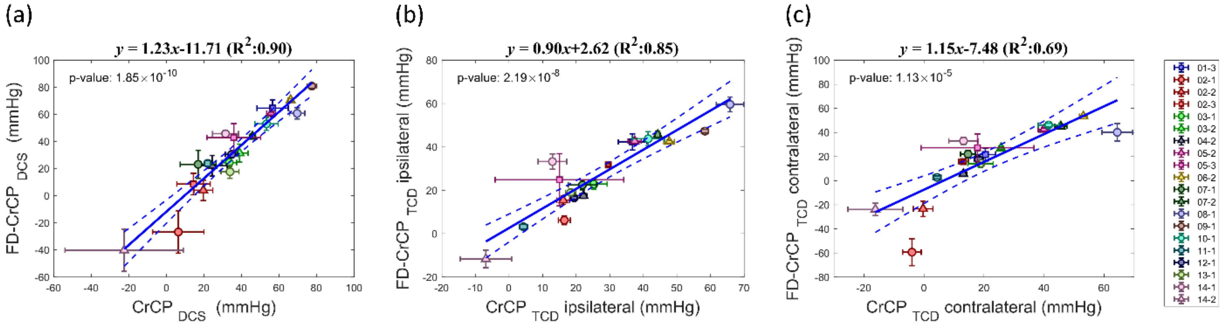

Figure S3. Comparing FD-CrCP and linear-regression derived CrCP: a) DCS, b) TCD ipsilateral to the stroke, c) TCD contralateral to the stroke. Different subjects are labelled with different colors. Session 1 circle, session 2 triangle, session 3 square. Dashed lines mark the confidence interval of the linear regression.

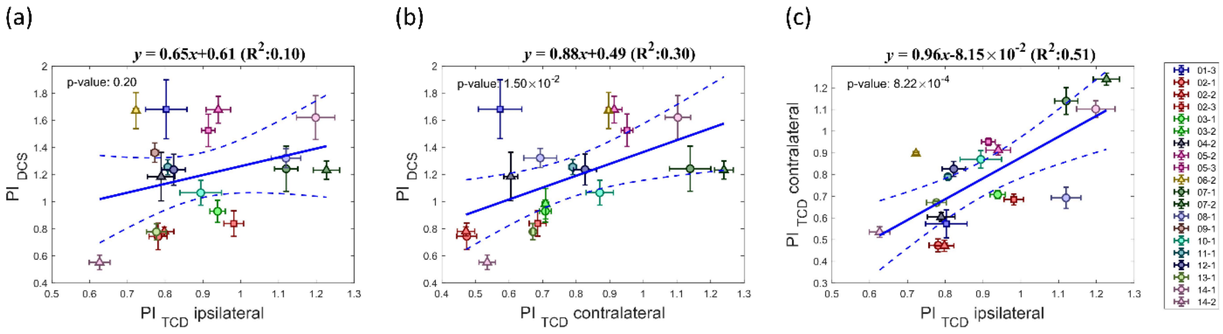

Figure S4. Scatterplots of pulsatility index mean and standard deviation of a) DCS vs. TCD ipsilateral to the stroke, b) DCS vs. TCD contralateral to the stroke. c) TCD ipsilateral vs. TCD contralateral to the stroke. Different subjects are labelled with different colors. Session 1 circle, session 2 triangle, session 3 square. Dashed lines mark the confidence interval of the linear regression.

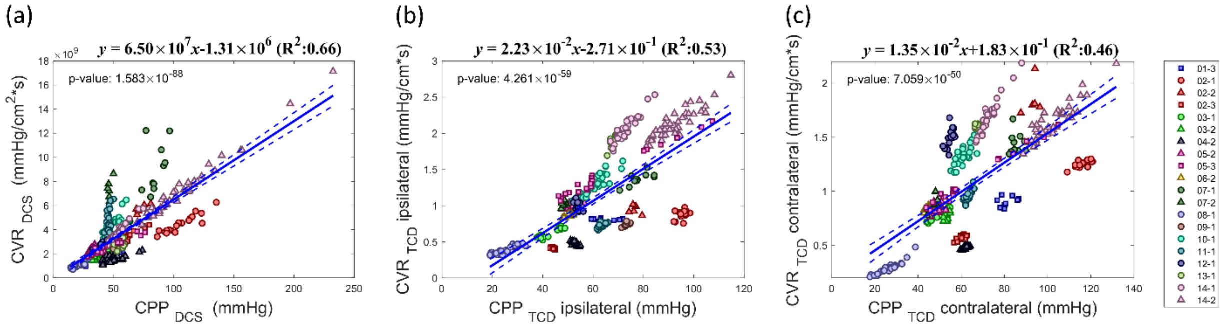

Figure S5. Scatterplots of CVR versus CPP of a) DCS, b) TCD ipsilateral to the stroke, c) TCD contralateral to the stroke. In each panel CPP was calculated as the difference between MAP and the CrCP estimated by the corresponding technology/side. Session 1 circle, session 2 triangle, session 3 square. Dashed lines mark the confidence interval of the linear regression.

2. Detailed CrCP data processing

After removing segment of data with motion artifacts, we identified the location of the heartbeats using arterial blood pressure. We used an algorithm to find systolic peaks and diastolic ends of each cardiac cycle.

Cardiac cycles do not have a constant length, outlier heartbeats deviating more than 4 standard deviation from the average were excluded. The remaining cycles were resampled and re-indexed to have the same number of data points in each cycle equal to the average length. Based on these indices, we resampled the g_2 curves by linearly interpolating neighboring g_2. In this way we obtained the same number of new g_2 curves for each cardiac cycle and maintain the 100 Hz resolution. The new g_2 curves at the same point of the cardiac cycle were averaged across 50 heartbeats, forming a series of g_2 per cardiac cycle. We then fit each g_2 curves in a series to derive a CBI cardiac waveform.
